# Retraction Note: Novel adjuvant nano-vaccine induced immune response against* Acinetobacter baumannii*

**DOI:** 10.1186/s13568-025-01923-4

**Published:** 2025-08-01

**Authors:** Tohid Piri-Gharaghie, Abbas Doosti, Seyed Abbas Mirzaei

**Affiliations:** 1 Department of Biology, Faculty of Basic Sciences, Shahrekord Branch, Islamic Azad University, Shahrekord, Iran; 2 Biotechnology Research Center, Shahrekord Branch, Islamic Azad University, Shahrekord, Iran; 3 Cellular and Molecular Research Center, Basic Health Sciences Institute, Shahrekord University of Medical Sciences, Shahrekord, Iran

**Retraction Note to: AMB Express (2023) 13:31** 10.1186/s13568-023-01531-0

The Editor-in-Chief has retracted this article. A number of image integrity issues were raised by a third party in this article (see below). The Authors were contacted for an explanation of concerns and raw images, however, they failed to provide a satisfactory explanation and raw images for all concerned figures.

The Editor-in-Chief therefore has lost confidence in these results and conclusions.

- Figure 3D CSNP (Before) appears to be similar to Figure 3D pDNA-CpG-CSNP (After).
- Figure 3D Free-pDNA-CSNP (Before) appears to be similar to Figure 3D Free-pDNA-CSNP (After).
- Figure 3D CSNP (After) appears to be similar to Figure 3D pDNA-CpG-CSNP (Before).
- Figure 6, agar plates for Blood—CSNP appear to be similar to agar plates, Liver—CSNP look similar.
- Figure 6, agar plates for Blood—PBS Liver appear to be similar to agar plates PBS.
- Figure 6, agar plates for Blood—CpG C274 appear to be similar to agar plates Lung—Free pDNA-CSNP.
- Figure 6 agar plates for Blood—pDNA-CpG C274 appear to be similar to agar plates Liver—CpG C274.
- In Additional File 3, the middle panel appears to be similar to the middle part of the left panel.

Abbas Doosti disagrees with this retraction. None of the remaining authors have responded to correspondence from the Publisher about this retraction.

